# Temporal validation of the MMCD score to predict kidney replacement therapy and in-hospital mortality in COVID-19 patients

**DOI:** 10.1186/s12882-023-03341-9

**Published:** 2023-10-04

**Authors:** Vanessa das Graças José Ventura, Polianna Delfino Pereira, Magda Carvalho Pires, Alisson Alves Asevedo, Alzira de Oliveira Jorge, Ana Carolina Pitanga dos Santos, André Soares de Moura Costa, Angélica Gomides dos Reis Gomes, Beatriz Figueiredo Lima, Bruno Porto Pessoa, Christiane Corrêa Rodrigues Cimini, Claudio Moisés Valiense de Andrade, Daniela Ponce, Danyelle Romana Alves Rios, Elayne Crestani Pereira, Euler Roberto Fernandes Manenti, Evelin Paola de Almeida Cenci, Felício Roberto Costa, Fernando Anschau, Fernando Graça Aranha, Flavia Maria Borges Vigil, Frederico Bartolazzi, Gabriella Genta Aguiar, Genna Maira Santos Grizende, Joanna d’Arc Lyra Batista, João Victor Baroni Neves, Karen Brasil Ruschel, Letícia do Nascimento, Lucas Moyses Carvalho de Oliveira, Luciane Kopittke, Luís César de Castro, Manuela Furtado Sacioto, Marcelo Carneiro, Marcos André Gonçalves, Maria Aparecida Camargos Bicalho, Mônica Aparecida da Paula Sordi, Natália da Cunha Severino Sampaio, Pedro Gibson Paraíso, Rochele Mosmann Menezes, Silvia Ferreira Araújo, Vivian Costa Morais de Assis, Katia de Paula Farah, Milena Soriano Marcolino

**Affiliations:** 1 Medical School and University Hospital, Universidade Federal de Minas Gerais, Av. Professor Alfredo Balena, 190, Belo Horizonte, Brazil; 2 Department of Internal Medicine, Medical School, Universidade Federal de Minas Gerais, Av. Professor Alfredo Balena, 190, Belo Horizonte, Brazil; 3 Institute for Health Technology Assessment (IATS/ CNPq), R. Ramiro Barcelos, 2359, Porto Alegre, Brazil; 4 Department of Statistics, Universidade Federal de Minas Gerais, Av. Presidente Antônio Carlos, 6627, Belo Horizonte, Brazil; 5 Universidade Federal Dos Vales Do Jequitinhonha E Mucuri (UFVJM), R. Cruzeiro, 01. , Teófilo Otoni, Minas Gerais Brazil; 6 Hospital Risoleta Tolentino Neves, R. das Gabirobas, 01, Belo Horizonte, Brazil; 7 Faculdade de Ciências Médicas de Minas Gerais, Al. Ezequiel Dias, 275, Belo Horizonte, Minas Gerais Brazil; 8 Hospitais da Rede Mater Dei, Av. Do Contorno, 9000, Belo Horizonte, Brazil; 9 Hospital Metropolitano Odilon Behrens, R. Formiga, 50, Belo Horizonte, Brazil; 10 Hospital Júlia Kubitschek, Av. Professor Alfredo Balena, 190, Belo Horizonte, Brazil; 11 Hospital Santa Rosália, R. Do Cruzeiro, 01, Teófilo Otoni, Brazil; 12 Computer Science Department, Universidade Federal de Minas Gerais, Av. Presidente Antônio Carlos, 6627, Belo Horizonte, Brazil; 13 Botucatu Medical School, Universidade Estadual Paulista “Júlio de Mesquita Filho”, Av. Prof. Mário Rubens Guimarães Montenegro, Botucatu, Brazil; 14 Hospital São João de Deus (Fundação Geraldo Correa), R. Do Cobre, 800, Divinópolis, Brazil; 15 Hospital SOS Cárdio, Rod. SC-401, 121, Florianópolis, Brazil; 16 Hospital Mãe de Deus, R. José de Alencar, 286, Porto Alegre, Brazil; 17 Hospital Universitário Canoas, Av. Farroupilha, 8001, Canoas, Brazil; 18 Hospital Nossa Senhora da Conceição, Av. Francisco Trein, 326, Porto Alegre, Brazil; 19 Hospital Metropolitano Doutor Célio de Castro, R. Dona Luiza, 311, Belo Horizonte, Brazil; 20 Hospital Santo Antônio, Pç. Dr. Márcio Carvalho Lopes Filho, 501, Curvelo, Brazil; 21 Universidade José Do Rosário Vellano (UNIFENAS), R. Boaventura, 50, Belo Horizonte, Brazil; 22 Santa Casa de Misericórdia de Belo Horizonte, Av. Francisco Sales, 1111, Belo Horizonte, Brazil; 23 Medical School, Universidade Federal da Fronteira Sul, SC-484 Km 02, Chapecó, Brazil; 24 Hospital Universitário de Santa Maria, Av. Roraima, 1000, Prédio 22, Santa Maria, Brazil; 25 Hospital Universitário Ciências Médicas de Minas Gerais, R. Dos Aimorés, 2896, Belo Horizonte, Brazil; 26 Hospital Bruno Born, Av. Benjamin Constant, 881, Lajeado, Brazil; 27 Hospital Santa Cruz, R. Fernando Abott, 174, Santa Cruz Do Sul, Brazil; 28 Hospital João XXIII, Av. Professor Alfredo Balena, 400, Belo Horizonte, Brazil; 29 Hospital Eduardo de Menezes, R. Dr. Cristiano Rezende, 2213, Belo Horizonte, Brazil; 30 Orizonti Instituto de Saúde E Longevidade, Av. José Do Patrocínio Pontes, 1355, Belo Horizonte, Brazil; 31 Hospital Semper, Al. Ezequiel Dias, 389, Belo Horizonte, Brazil; 32 Telehealth Center, University Hospital, Universidade Federal de Minas Gerais, Av. Professor Alfredo Balena, 110, Belo Horizonte, Brazil

**Keywords:** COVID-19, Acute kidney injury, Kidney replacement therapy, Score predictive, Risk prediction, Mortality

## Abstract

**Background:**

Acute kidney injury has been described as a common complication in patients hospitalized with COVID-19, which may lead to the need for kidney replacement therapy (KRT) in its most severe forms. Our group developed and validated the MMCD score in Brazilian COVID-19 patients to predict KRT, which showed excellent performance using data from 2020. This study aimed to validate the MMCD score in a large cohort of patients hospitalized with COVID-19 in a different pandemic phase and assess its performance to predict in-hospital mortality.

**Methods:**

This study is part of the “Brazilian COVID-19 Registry”, a retrospective observational cohort of consecutive patients hospitalized for laboratory-confirmed COVID-19 in 25 Brazilian hospitals between March 2021 and August 2022. The primary outcome was KRT during hospitalization and the secondary was in-hospital mortality. We also searched literature for other prediction models for KRT, to assess the results in our database. Performance was assessed using area under the receiving operator characteristic curve (AUROC) and the Brier score.

**Results:**

A total of 9422 patients were included, 53.8% were men, with a median age of 59 (IQR 48–70) years old. The incidence of KRT was 8.8% and in-hospital mortality was 18.1%. The MMCD score had excellent discrimination and overall performance to predict KRT (AUROC: 0.916 [95% CI 0.909–0.924]; Brier score = 0.057). Despite the excellent discrimination and overall performance (AUROC: 0.922 [95% CI 0.914–0.929]; Brier score = 0.100), the calibration was not satisfactory concerning in-hospital mortality. A random forest model was applied in the database, with inferior performance to predict KRT requirement (AUROC: 0.71 [95% CI 0.69–0.73]).

**Conclusion:**

The MMCD score is not appropriate for in-hospital mortality but demonstrates an excellent predictive ability to predict KRT in COVID-19 patients. The instrument is low cost, objective, fast and accurate, and can contribute to supporting clinical decisions in the efficient allocation of assistance resources in patients with COVID-19.

**Supplementary Information:**

The online version contains supplementary material available at 10.1186/s12882-023-03341-9.

## Background

Acute kidney injury (AKI) has been described as a common complication in patients hospitalized with COVID-19, which may lead to the need for kidney replacement therapy (KRT) and mortality in its most severe forms. An umbrella review and meta-analysis identified an incidence rate of AKI 27.2% (95% confidence interval [CI]: 23.8–30.5%) in patients hospitalized with COVID-19, with an overall incidence of KRT between 2.2 and 7.7%, and an odds ratio of 5.24 (95% CI 3.96–6.93) for mortality among those who developed AKI [1]. The risk of an unfavorable outcome, such as progression to severe KRT requirement or death, is greater in vulnerable patients, such as the elderly and patients with comorbidities, especially diabetes, chronic kidney disease, and transplant recipients [2–4].

Predictive scores have been developed to estimate and risk stratify inpatients with COVID-19. Using a risk score to predict KRT requirement during hospital stay may be very helpful to establish kidney protection measures, determining which patients need more intensive monitoring of kidney function, and adding to care planning the multi-professional team [5, 6]. In this context, our group developed and validated the MMCD score, consisting of four criteria that are accessible in clinical practice and are quick and easy to apply: M (male sex), M (mechanical ventilation anytime during hospital stay), C (creatinine at hospital presentation), D (diabetes). To the best of our knowledge, it is still the only score to predict KRT among in-hospital COVID-19 patients published in a scientific journal [7]. It is also the only score calculator for this purpose published in the Dynamed medical summary [8]. The score showed outstanding discrimination and high overall performance using data from patients admitted in 2020 [7].

Other scores commonly used in intensive care have been tested to predict KTR among in-hospital patients, with poor results. A Brazilian study showed poor discrimination ability of the Simplified Acute Physiology Score 3 (SAPS3) and the Sequential Organ Failure Assessment (SOFA) to predict acute kidney injury and KRT requirement, with an area under the receiver operator characteristic curve (AUROC) of 0.590 (95% CI 0.507–0.674) and 0.667 (95% CI 0.591–0.743) [9].

Clearly, during the COVID-19 pandemic, there was a reduction in morbidity and mortality rates related to improvements in the management, the emergence of variants, and especially population immunization [10–12]. However, mortality related to COVID-19 and disease complications remain high in vulnerable groups and contrast the general feeling of control of the pandemic arising from a milder predominant variant and advances in the management and immunization of the population [13]. Thus, a new temporal validation of the MMCD score is warranted and required [5].

Therefore, this study aimed to perform a new temporal validation of the MMCD score in a large cohort of COVID-19 patients from a more recent pandemic phase, as well as to evaluate this score in the prediction of in-hospital mortality.

## Material and methods

The present study is part of the “Brazilian COVID-19 Registry”, a retrospective observational cohort, described in detail elsewhere [14, 15]. Temporal validation followed methodological rigor from Transparent Reporting of a Multivariable Prediction Model for Individual Prediction or Diagnosis (TRIPOD) and Prediction model Risk of Bias Assessment Tool (PROBAST) [5, 16].

### Study design

**Fig. 1:**
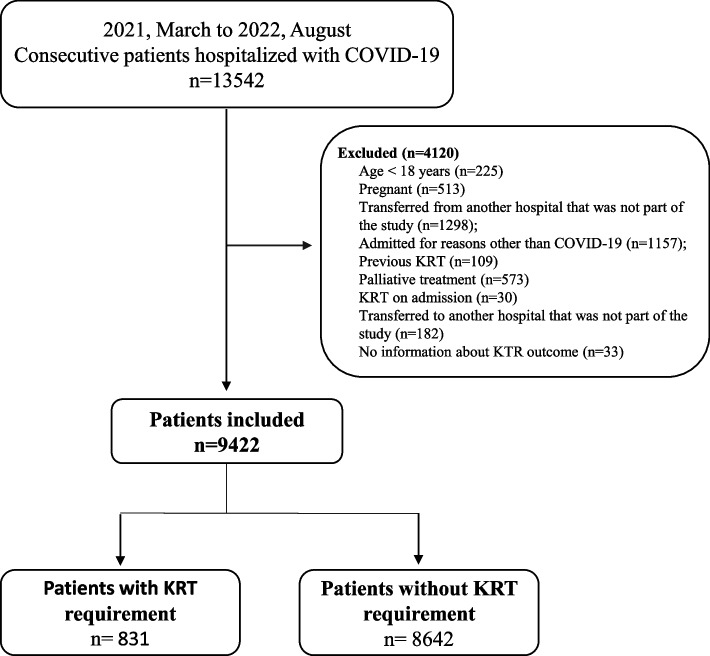
Flowchart of Brazilian patients included in the study. KRT: kidney replacement therapy

The study included consecutive adult patients (≥ 18 years old), with laboratory-confirmed COVID-19, admitted in one of the 25 participating hospitals (public, private, and philanthropic) from 12 cities in the Southeast and South of the country, from March 2021 to August 2022. Pregnant women, patients who were transferred from another hospital that was not part of the study or who manifested COVID-19 while admitted for other conditions, those undergoing palliative treatment, with a history of prior KRT or in KRT upon hospital presentation, and those who were transferred to another hospital that was not part of the study were excluded from the analysis (Fig. 1).

### Data collection

Trained researchers collected patient data from the medical records using the Research Data Capture (REDCap®) electronic platform, hosted at the Telehealth Center of the University Hospital of the *Universidade Federal de Minas Gerais* [17, 18]. Sociodemographic, clinical, and laboratory data were collected during the hospital stay, particularly on hospital presentation and admission to the ICU (if required). In addition, information on medications, interventions, and outcomes was collected from admission to hospital discharge or death, including the start and end time of invasive mechanical ventilation (MV) and KRT. Each patient was represented in the database by an individual code that considered the hospital of admission. In order to ensure data reliability, a detailed collection manual was prepared, everyone involved was extensively trained, and a data verification code was developed using R. In the face of any doubts about the consistency of the data, the researchers contacted the center coordinators to confirm or correct the information.

### The predictive MMCD score

The MMCD was developed and validated as a predictive score for KRT requirement in patients with COVID-19, based on four simple and objective criteria: the need for **m**echanical ventilation anytime during the hospital stay, **m**ale sex, serum **c**reatinine at the hospital admission, and **d**iabetes [7]. The need for MV anytime during the hospital stay was the variable with the highest score in risk score (11 points). Serum creatinine levels were categorized according to the SOFA, and higher creatinine levels received higher scores (creatinine between 1.2–2.0—1 point; 2.0 – 3.5—2 points, 3.5—5.0—4 points and ≥ 5.0—10 points). Male sex and the presence of diabetes mellitus represented 1 point each. The sum of prediction scores ranges from 0 to 23, with a high score indicating an increased risk of KRT. According to predicted probabilities, three risk groups were defined: low (score 0–10, KRT 0.4%), high risk (score 11–14, KRT 32.8%), and very high risk (score 15– 23, KRT 68.0%) [7].

### Comparison with other prediction models

In order to identify other prediction models to predict KRT, we performed a literature search on Medline, medRxiv and BioRxiv and SSRN, with no language or date restrictions, using the search terms “COVID” combined with “renal replacement therapy”. The last search was performed on August 15, 2023.

From the set of identified scores, those with predictors available within our database and had accessible methods for calculation were selected.

The search identified two studies [19, 20]. Vaid et al. (2021) assessed the combined outcome of KRT or death and used variables that were not available in our database (red cell distribution width, alkaline phosphatase, and anion gap), so it was not included in the analyses [19].

Therefore, the study by Franca et al. (2023) was the only one identified which met our analysis criteria. The authors developed and validated a predictive model for KRT in a multicenter cohort of adult patients with COVID-10, admitted to ICUs in Brazil with ventilatory support requirement, from February 2020 to May 2022. Statistical and machine-learning methods were used for classification. Model comparisons were performed using AUROC and the Brier score [20].

### Outcomes

The primary outcome was the KRT requirement during hospital stay. The secondary outcome was in-hospital mortality.

### Statistical analysis

In the descriptive analyses, continuous data were presented by medians and interquartile ranges (IQR), while categorical data were described as absolute numbers and proportions. Comparison between patient groups was performed using Wilcoxon rank sum test for continuous variables, and Fisher's exact test for categorical variables. The significance level was set at a two-tailed *p*-value ≤ 0.05.

Multiple imputation by chained equations (MICE) was used to handle missing values. The imputation technique included all variables with up to 30% missing values. The results of 10 imputed datasets, each with 10 iterations, were combined following Rubin's rules [21]. The KRT requirement was not used as a predictor in MICE. The least absolute shrinkage and selection operator (LASSO) linear regression method was used for continuous predictors, LASSO logistic regression for two levels, and polytomous regression for more than two level categorical variables.

Model's discrimination was assessed by the AUROC with 95% confidence intervals (CI). The value usually ranges from 0.5, meaning the model has no predictive power, to 1.0, which means the model predicts the classes perfectly. Thus, the closer to 1, the better the predictive power of the model, with values between 0.7 and 0.8 considered acceptable, between 0.8 and 0.9 excellent, and above 0.9 outstanding [22].

In addition to discrimination, a good predictive model needs to be properly calibrated, that is, agreement between observed and predicted events [23, 24]. The plot with predicted probability against observed probability described the model’s calibration, testing intercept equals zero and slope equals one.

Finally, the accuracy of the predictive model was evaluated by the Brier score. The score varies between 0 and 1, with values close to 0 related to the proximity between predictions and reality, indicating better model performance [25].

Statistical analysis was performed with R software (version 4.0.2), using the gtsummary, mice, pROC, rms, rmda, and psfmi packages.

#### Machine Learning analytics

In addition, we replicated the machine learning-based analysis described by Franca et al. (2023), et al., in our database using the random forest classifier and the same variables used by the authors [20]. For this analysis, we employed the stratified fivefold cross-validation process. In this process, the dataset was divided into 5 parts, maintaining the class proportions, known as “folds”. The process was repeated 5 times. In each iteration, one of the folds was selected as the test set, while the other 4 folds were used to compose the training and validation set. The training was used for model learning, while the validation was employed for parameter tuning. Finally, the results of the AUROC and its respective 95% confidence interval and Brier score were reported on the test set.

For this analysis, we considered all patients included in the present study and a subgroup analysis of patients who were admitted to intensive care, as Franca et al. (2023) [20]. Additionally, we performed the analysis replacing the variable MV on admission, which is the variable used by Franca et al. (2023) with MV anytime during hospital stay, which is the variable included in the MMCD score [7].

### Ethics approval

This study was approved by the Brazilian National Commission for Research Ethics (*Comissão Nacional de Ética em Pesquisa*, approval number CAAE 30350820.5.0000.0008) and by the ethics committee of each individual participant institution. Individual informed consent term was waived by the Brazilian National Commission for Research Ethics and the institutions’ ethics committees due to the pandemic situation and analysis of deidentified data, based on chart review only.

## Results

A total of 9422 patients were included, 53.8% were men, with a median age of 59 (IQR 48–70) years old. The incidence of AKI with KRT requirement was 8.8% and in-hospital mortality was 18.01%. Table 1 shows demographic, clinical characteristics, and patient outcomes. The most prevalent comorbidities were hypertension (52.0%), diabetes (25.5%), and obesity (18.6%). Overall, 32.2% of the patients required admission to an ICU and 24.0% required invasive MV (Table 1).

**Table 1:** Demographic, clinical characteristics, and outcomes of the patients hospitalized with COVID-19, considering the need for kidney replacement therapy, 2021/2022

**Variables**	**Overall**^a^ (*n* = 9422)	**KRT**^a^ (*n* = 831)	**No KRT**^a^ (*n* = 8591)	***p*-value**^**2**^
Age (years)	59 (48–70)	64 (56–73)	59 (47–70)	< 0.001
Male	5100 (53.8%)	522 (62.8%)	4548 (52.9)	< 0.001
*Comorbidities*				
Hypertension	4928 (52.0%)	532 (64.0%)	4368 (50.8%)	< 0.001
Coronary artery disease	389 (4.1%)	45 (5.4%)	344 (4.0%)	0.055
Heart failure	449 (4.7%)	39 (4.7%)	408 (4.7%)	> 0.999
Stroke	251 (2.6%)	15 (1.8%)	235 (2.7%)	0.140
Asthma	541 (5.7%)	40 (4.8%)	494 (5.8%)	0.307
COPD	469 (5.0%)	47 (5.7%)	426 (5.0%)	0.361
Diabetes mellitus	2412 (25.5%)	299 (36.0%)	2101 (24.5%)	< 0.001
Obesity	1768 (18.7%)	207 (24.9%)	1547 (18.0%)	< 0.001
Cirrhosis	27 (0.3%)	5 (0.6%)	22 (0.3%)	0.084
Chronic kidney disease	295 (3.1%)	58 (7.0%)	233 (2.7%)	< 0.001
HIV infection	65 (0.7%)	8 (1.0%)	60 (0.7%)	0.387
Cancer	305 (3.2%)	29 (3.5%)	279 (3.2%)	0.683
Previous transplantation	75 (0.8%)	19 (2.3%)	54 (0.6%)	< 0.001
Lifestyle habits				
Illicit drugs	59 (0.6%)	2 (0.2%)	56 (0.7%)	0.239
Alcoholism	552 (5.8%)	41 (4.9%)	512 (6.0%)	0.247
Current smoker	399 (4.2%)	35 (4.2%)	363 (4.2%)	> 0.999
*Admission data*				
Glasgow coma score < 15	452 (4.8%)	39 (4.7%)	408 (4.7%)	> 0.999
Systolic blood pressure (mmHg)	125 (116–140)	130 (120–140)	124 (116- 140)	0.005
Heart rate (bpm1)	85 (76–95)	88 (80–97)	85 (75–95)	< 0.001
Respiratory rate (bpm2)	20 (18–24)	24 (20–28)	20 (18–24)	
SpO2/FiO2	350 (290.6–433.3)	339.3 (216.3–423.8)	350.0 (293.8, 438.1)	
*Outcomes*				
Admission to the ICU	3042 (32.1%)	810 (97.5%)	2220 (25.9%)	< 0.001
Invasive MV	2272 (24.0%)	779 (93.7%)	1482 (17.3%)	< 0.001
In-hospital mortality	1705 (18.0%)	675 (81.2%)	1031 (12.0%)	< 0.001

*COPD* Chronic obstructive pulmonary disease, *HIV* Human immunodeficiency virus, *ICU* Intensive care unit, *KRT* Kidney replacement therapy, *MV* Mechanical ventilation

^a^Values in frequencies (percentage) or medians (interquartile range)

^2^Wilcoxon rank sum test; Fisher's exact test

Patients with KRT requirement were mostly men and older, had higher prevalence of hypertension, diabetes mellitus, obesity, chronic kidney disease, and a history of previous transplantation when compared to those who did not require KRT. They also had a higher frequency of ICU admission, VM requirement, and mortality (Table 1). Considering the patients who died when compared to those who did not, again the majority were men and older, with the same profile of comorbidities than the patients with KRT requirement, adding a higher prevalence of cancer. With regards to the outcomes, they had a higher rate of ICU admission, MV, and KRT requirement (Table S1).

Clinical symptoms and laboratory features in overall, patients with KRT requirement or patients who died are presented in the supplemental material (Table S2-S3).

### Model performance

**Fig. 2:**
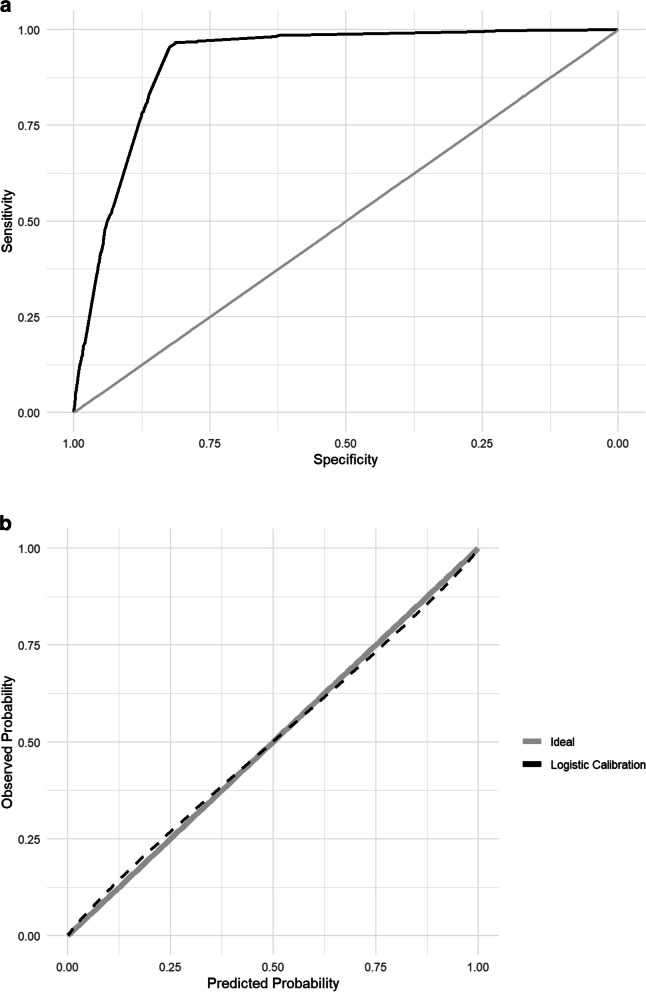
**a** Area under the receiver operating characteristic curve for the validation of MMCD score to outcome kidney replacement therapy. **b** Calibration for the validation of MMCD score to outcome kidney replacement therapy

**Fig. 3:**
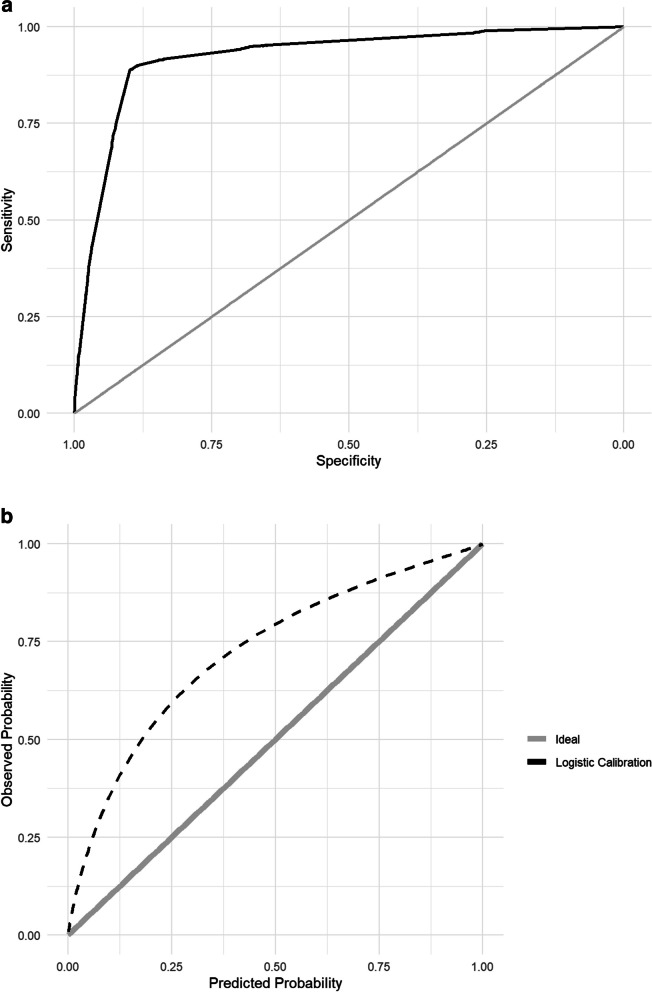
**a** Area under the receiver operating characteristic curve for the validation of MMCD score to in-hospital mortality. **b** Calibration for the validation of MMCD score to in-hospital mortality

The MMCD score had outstanding discrimination (AUROC: 0.916 [95% CI 0.909–0.924]), overall performance (Brier score = 0.057 and calibration (slope = 0.916, intercept = -0.005, *p*-value = 0.0001)) (Fig. 2a and b) to predict KRT. Additionally, the MMCD score showed excellent discrimination (AUROC: 0.922 [95% CI 0.914–0.929]) and overall performance (Brier score = 0.100), but calibration (slope = 0.886; intercept = 1.354; *p*-value = 0.000) was not satisfactory to predict in-hospital mortality (Fig. 3a and b).

### Comparison with other prediction models

When the random forest model by Franca et al. (2023) was applied to our database, the discrimination was acceptable (AUROC: 0.71 [95% CI 0.69–0.73]) and Brier score 0.08 [20]. Considering the subgroup analysis with only patients admitted to the intensive care unit, the model's predictive performance was weak (AUROC: 0.66 [95% CI 0.64–0.68]) and Brier score 0.19. The most important predictors in both contexts were creatinine, urea, and platelets upon hospital presentation or ICU admission, respectively, and sex. Hypertension, diabetes, and MV were not among the most important predictors (Table S4).

Subsequently, in the analysis which replaced the variable MV on admission included in the random forest model by Franca et al. (2023) by MV anytime during hospital stay, which is included in the MMCD score, the random forest model showed outstanding discrimination (AUROC: 0.92 [0.91–0.93]) and Brier score 0.06 [20].

## Discussion

In this study, from a robust cohort of COVID-19 patients from 25 Brazilian hospitals, admitted through the second and third pandemic waves in Brazil, 8.8% required KRT and 18.0% died. The MMCD score presented outstanding discrimination (AUROC: 0.916 [95% CI 0.909–0.924]), and overall performance (Brier score = 0.057) in KRT requirement prediction. With regards to in-hospital mortality, the score had excellent discrimination (AUROC: 0.922 [95% CI 0.914–0.920]) and overall performance (Brier score = 0.100), but not satisfactory calibration.

Special attention to kidney outcomes is important because, despite vaccination, AKI is still a common and serious complication, being considered an independent risk factor for mortality. Many of these patients require KRT, increasing the resources needed for care and evolving greater morbidity and mortality. Measures to prevent the occurrence or progression to a more severe progression of AKI are fundamental [26, 27]. Thus, early prognostic assessment and risk stratification measures that support clinical decisions in allocating appropriate resources and early interventions to each patient have the potential to enhance personalized hospital care and to help reduce morbidity and mortality rates [28]. Among the precocious preventive measures, care with water balance, glycemic control, and the indication and monitoring of potentially nephrotoxic medications can be highlighted. In addition, the application of the MMCD score could help in the decision to early transfer patients with a higher probability of unfavorable evolution to referral centers, if the hospital where the patient is located does not have a dialysis service [6, 29]. In this context, closer monitoring is necessary in patients with MMCD score greater than or equal to 11, especially if greater than or equal to 14.

To our best knowledge, the MMCD is the only score designed to predict the risk of KRT in patients with COVID-19 [7]. In this new temporal validation, the score maintained excellent discrimination and overall performance. New temporal validations of a score are of utmost importance to ensure that the score maintains its discriminatory power in a constantly changing population [5]. An example of the importance of temporal validation of a score was the recent removal of the well-known Framingham score from the study page [30]. The Framingham score from 2008 was developed with data from a large American cohort and aimed to predict cardiovascular disease in ten years. For many years it was recognized as a very helpful clinical assessment tool, but there is doubt about whether it remains valid today, 20 years after the publication of its results [2, 31]. In this context, validating the predictive score of outcomes related to COVID-19, in different phases of the pandemic, is crucial, due to changes in disease profile and vaccination rate.

The MMCD score obtained a significantly higher discrimination capacity than the model by Franca et al. (2023), using fewer and simpler variables, therefore with greater potential for applicability in clinical practice [7, 20]. The most important predictor in the MMCD score was MV anytime during hospital stay. The relationship between MV and AKI has been widely recognized in critically ill patients before COVID-19 [32]. The mechanisms involved are complex, multifactorial, and there are still some gaps in knowledge. It is believed that they include hemodynamic factors related to reduced kidney perfusion secondary to positive intrathoracic pressure, selective renal vasoconstriction by sympathetic stimulation, and endothelial damage resulting from the release of pro-inflammatory mediators by the lungs [32–34]. In patients with severe COVID-19, it is common to develop acute respiratory distress syndrome (ARDS), a condition often treated with higher positive end-expiratory pressure (PEEP), which increases intrathoracic pressure and can result in increased renal venous pressure, reduced kidney perfusion, and glomerular filtration [35]. In addition, increased intrathoracic pressure can also interfere with right ventricular function, reducing venous return and causing secondary kidney congestion [29]. Renal hemodynamics abnormalities are even more accentuated due to renal vasoconstriction and activation of the renin-angiotensin system secondary to the increase in sympathetic tone related to MV [29]. Furthermore, severe COVID-19-related severe lung injury can lead to release of cytokines, chemokines, vasoactive agents, and damage-associated molecular patterns (DAMPs) into the systemic circulation [35]. Kidney effects include cell damage, interstitial edema, and microthrombosis that may mediate AKI [35].

The model by Franca et al. (2023) included MV upon admission among the predictors, unlike the MMCD, which considered MV anytime during hospital stay [7, 20]. When we replaced the variable MV on admission by MV anytime during hospital stay, model discrimination increased significantly, reaching outstanding results (AUROC: 0.92 [95%CI 0.91–0.93]). This highlights the need of a dynamic analysis, considering MV anytime during hospital stay.

In the present study, our group tried to validate the MMCD score for predicting KRT and in-hospital mortality, considering that the use of a single instrument to predict two severe outcomes would facilitate clinical applicability, especially in contexts with high demand and severity of patients, such as an emergency room, or with limited resources. However, despite the excellent discrimination and overall performance, the calibration curve showed that the MMCD score overestimated mortality. Many validation studies of predictive scores present performance only with discrimination, especially AUROC, omitting calibration data [36]. The analysis carried out demonstrates the importance of a complete evaluation of a predictive modelfor the correct prediction and reliability of the clinical application of the score in clinical practice [37, 38].

### Strengths and limitations

The MMCD score is a simple instrument, low cost, uses objective parameters, obtained routinely by the institutions. The rigorous methodology in the data collection and auditing generates reliability of the results. Furthermore, the sample with nearly 10,000 patients from hospitals distributed in different Brazilian geographic regions increases the probability of the generalization of results to the Brazilian population. Thus, the MMCD has a great potential to contribute to the assertive management of care resources and consequently to the reduction of kidney complications and thus reduction of morbidity and mortality.

Another important point is that the MMCD uses data from hospital admission and MV anytime during hospital stay. The use of unique admission data can deviate a patient's course from predictions due to post-admission adverse events. In this sense, the use of the need for MV makes the predictive model more continuous and the prediction more appropriate.

Last but not the least, the MMCD validation included patients from the second and third wave of the COVID-19 pandemic, phases of the pandemic that are very different from each other and concerning the moment of derivation and validation of the MMCD score. The second wave was predominantly affected by the delta variant, which had the worst incidence and mortality rates in Brazil. The omicron variant predominated in the third wave in which immunization had already been carried out in the most vulnerable individuals and was progressing more quickly throughout the entire population [10–12].

Even with these multiple strengths, the present study has limitations that should be addressed. The lack of baseline creatinine previous to COVID-19 and data from patient diuresis prevented the assessment of AKI [6]. It is not possible to know whether the increase in serum creatinine on admission is an AKI, a chronic kidney disease (CKD), or even an acute-on-CKD, but this had no effect in the predictive model, which showed excellent discrimination and calibration. Despite being important data, it is common for information on previous creatinine to be absent in patients admitted to hospitals, making the application of a risk score capable of predicting KRT even more important. Another point to be highlighted is the lack of data regarding the specific indications for the initiation of KRT. The time and indication for starting KRT were determined by each service based on the assessment of each patient, which may cause variation in rates. However, as the KDIGO guidelines are widely recognized and implemented internationally, the small variation might have not impacted the results [6, 39]. Furthermore, as recognized for any predictive score, the application of the MMCD to other populations requires news geographic validations in the face of the changes in the disease profile [5].

### Perspectives

In a similar manner that happens with other risk scores, periodical adjustments should also be considered for the MMCD score. Considering the practicality and quality of the MMCD score, performance assessment in other contexts, such as sepsis, would be interesting. These topics may be the next steps of our study.

## Conclusion

This study temporally validated the MMCD score, a risk prediction score for KRT. MMCD score demonstrates an excellent predictive ability to KRT in COVID-19 patients hospitalized in 2021/2022. The instrument is low cost, objective, fast and accurate, and can contribute to supporting clinical decisions in the efficient allocation of care resources in patients with COVID-19. Regarding in-hospital mortality, despite the excellent discrimination, the score overestimates the outcome, therefore, it is not suitable for the prediction of this outcome.

### Supplementary Information

**Additional file 1: Table S1.** Demographic, clinical characteristics, and outcomes of the patients hospitalized with COVID-19, considering death and discharge, 2021/2022.

**Additional file 2: Table S2.** Clinical manifestations and laboratory findings of the patients hospitalized with COVID-19, considering the need for kidney replacement therapy, 2021/2022.

**Additional file 3: Table S3.** Clinical manifestations and laboratory findings of the patients hospitalized with COVID-19, considering death and discharge, 2021/2022.

**Additional file 4:** **Table S4:** Features' importance and contribution to the final model.

**Additional file 5:** **Table S5.** TRIPOD checklist for transparent reporting on a multivariable prognostic model.

## Data Availability

The data generated or analyzed during this study are included in this article and its supplementary information files. The corresponding author is available to provide additional data regarding this manuscript upon request.

## Abbreviations

AKI: Acute kidney injury
ARDS: Acute respiratory distress syndrome
AUROC: Area under the receiver operating characteristic
BPM1: Beat per minute
BPM2: Breaths per minute
CI: Confidence interval
CKD: Chronic kidney disease
COVID-19: Coronavirus disease 19
HIV: Human immunodeficiency virus
ICU: Intensive care unit
IQR: Interquartile range
KDIGO: Kidney Disease: Improving Global Outcomes
KRT: Kidney replacement therapy
LASSO: Least absolute shrinkage and selection operator
MMCD: Mechanical ventilation anytime during hospital stay, Male, Creatinine, Diabetes
MV: Mechanical ventilation
PROBAST: Prediction model Risk of Bias Assessment Tool
REDCap: Research Electronic Data Capture
SAPS3: Simplified Acute Physiology Score 3
SOFA: Sequential Organ Failure Assessment
TRIPOD: Transparent Reporting of a Multivariable Prediction Model for Individual Prediction or Diagnosis
