# Supplementary material for: Temporal validation of the MMCD score to predict kidney replacement therapy and in-hospital mortality in COVID-19 patients
Source: BMC Nephrol. 2023 Oct 4;24:292. doi: 10.1186/s12882-023-03341-9 (PMC10552198; doi:10.1186/s12882-023-03341-9)
Supplement: Supplementary file 1 — Additional file 1: Table S1. Demographic, clinical characteristics, and outcomes of the patients hospitalized with COVID-19, considering death and discharge, 2021/2022. [file 12882_2023_3341_MOESM1_ESM.docx]

**Table S1.** Demographic, clinical characteristics, and outcomes of the patients hospitalized with COVID-19, considering death and discharge, 2021/2022.

| **Variables** | **Overall^1^**  (n=9422) | **Death**^1^ (n=1706) | **Discharge^1^** (n=7715) | **p-value^2^** |
| --- | --- | --- | --- | --- |
| Age (years) | 59 (48-70) | 66 (57-75) | 57 (46-69) | <0.001 |
| Male | 5069 (53.8%) | 956 (56.0%) | 4113 (53.3%) | 0.044 |
| *Comorbidities* |  |  |  |  |
| Hypertension | 4,899 (52.0%) | 1064 (62.4%) | 4899 (52.0%) | <0.001 |
| Coronary artery disease | 389 (4.1%) | 98 (5.7%) | 291 (3.8%) | <0.001 |
| Heart failure | 447 (4.7%) | 110 (6.4%) | 337 (4.4%) | <0.001 |
| Stroke | 250 (2.7%) | 48 (2.8%) | 202 (2.6%) | 0.618 |
| Asthma | 534 (5.7%) | 85 (5.0%) | 449 (5.8%) | 0.184 |
| COPD | 473 (5.0%) | 111 (6.5%) | 362 (4.7%) | 0.003 |
| Diabetes mellitus | 2400 (25.5%) | 597 (35.0%) | 1803 (23.4%) | <0.001 |
| Obesity | 1754 (18.6%) | 385 (22.6%) | 1369 (17.7%) | <0.001 |
| Cirrhosis | 27 (0.3%) | 8 (0.5%) | 19 (0.2%) | 0.132 |
| Chronic kidney disease | 291 (3.1%) | 88 (5.2%) | 203 (2.6%) | <0.001 |
| HIV infection | 68 (0.7%) | 14 (0.8%) | 54 (0.7%) | 0.635 |
| Cancer | 308 (3.3%) | 74 (4.3%) | 234 (3.0%) | 0.008 |
| Previous transplantation | 73 (0.8%) | 22 (1.3%) | 51 (0.7%) | 0.013 |
| Lifestyle habits |  |  |  |  |
| Illicit drugs | 58 (0.6%) | 7 (0.4%) | 51 (0.7%) | 0.304 |
| Alcoholism | 553 (5.9%) | 90 (5.3%) | 463 (6.0%) | 0.279 |
| Current smoker | 398 (4.2%) | 73 (4.3%) | 325 (4.2%) | 0.894 |
| *Admission data* |  |  |  |  |
| Glasgow coma score < 15 | 447 (4.7%) | 117 (6.9%) | 330 (4.3%) | <0.001 |
| Systolic blood pressure (mmHg) | 125 (116-140) | 125 (112-140) | 125 (117-140) | 0.977 |
| Heart rate (bpm1) | 85 (76-95) | 87 (78-98) | 85 (75-95) | <0.001 |
| Respiratory rate (bpm2) | 20 (18-24) | 23 (20-28) | 20 (18-24) | <0.001 |
| SpO2/FiO2 | 350.0 (290.6-433.3) | 353.6 (296.9-438.1) | 306.2 (160.0-414.3) | <0.001 |
| *Outcomes* |  |  |  |  |
| Admission to the ICU | 3030 (32.2%) | 1511 (88.6%) | 1519 (19.7%) | <0.001 |
| Invasive MV | 2261 (24.0%) | 1509 (88.5%) | 752 (9.7%) | <0.001 |
| KRT | 831 (8.8%) | 675 (39.6%) | 156 (2.0%) | <0.001 |

^1^Values in frequencies (percentage) or medians (interquartile range). ^2^Wilcoxon rank sum test; Fisher's exact test. COPD: chronic obstructive pulmonary disease; HIV: human immunodeficiency virus; ICU: intensive care unit; KRT: kidney replacement therapy; MV: mechanical ventilation.
