# Supplementary material for: Temporal validation of the MMCD score to predict kidney replacement therapy and in-hospital mortality in COVID-19 patients
Source: BMC Nephrol. 2023 Oct 4;24:292. doi: 10.1186/s12882-023-03341-9 (PMC10552198; doi:10.1186/s12882-023-03341-9)
Supplement: Supplementary file 2 — Additional file 2: Table S2. Clinical manifestations and laboratory findings of the patients hospitalized with COVID-19, considering the need for kidney replacement therapy, 2021/2022. [file 12882_2023_3341_MOESM2_ESM.docx]

**Table S2.** Clinical manifestations and laboratory findings of the patients hospitalized with COVID-19, considering the need for kidney replacement therapy, 2021/2022.

| **Variables** | **Overall^1^**  (n=9422) | **KRT^1^**  (n=831) | **No KRT^1^** (n=8591) | **p-value2** |
| --- | --- | --- | --- | --- |
| *Symptoms* |  |  |  |  |
| Adynamia | 2027 (21.5%) | 193 (23.2%) | 1834 (21.3%) | 0.216 |
| Ageusia | 826 (8.8%) | 74 (8.9%) | 752 (8.8%) | 0.898 |
| Anosmia | 913 (9.7%) | 85 (10.2%) | 828 (9.6%) | 0.580 |
| Headache | 2051 (21.8%) | 146 (17.6%) | 1905 (22.2%) | 0.002 |
| Rhinorrhea | 1185 (12.6%) | 98 (11.8%) | 1087 (12.7%) | 0.511 |
| Diarrhea | 1499 (15.9%) | 137 (16.5%) | 1362 (15.9%) | 0.620 |
| Dyspnea | 6229 (66.1%) | 569 (68.5%) | 5660 (65.9%) | 0.135 |
| Sore throat | 954 (10.1%) | 86 (10.3%) | 868 (10.1%) | 0.810 |
| Fever | 4850 (51.5%) | 404 (48.6%) | 4446 (51.8%) | 0.088 |
| Hyporexia | 1430 (15.2%) | 148 (17.8%) | 1282 (14.9%) | 0.029 |
| Myalgia | 3081 (32.7%) | 245 (29.5%) | 2836 (33.0%) | 0.040 |
| Náusea / Vomiting | 1299 (13.8%) | 116 (14.0%) | 1183 (13.8%) | 0.874 |
| Dry cough | 6383 (67.7%) | 540 (65.0%) | 5843 (68.0%) | 0.080 |
| *Admission data* |  |  |  |  |
| Hemoglobin (g/dL) | 13.3 (12.1-14.4) | 13.3 (12.1-14.6) | 13.3 (12.2-14.4) | 0.508 |
| White blood cell count (x109/L) | 7530 (5600- 10080) | 7240 (5300- 9830) | 7550 (5620- 10100) | 0.060 |
| Neutrophilis (x109/L) | 5690 (4004- 8057) | 5840 (3941-8004) | 5,-680.0 (4,008.0, 8,057.5) | 0.953 |
| Lymphocytes (x109/L) | 978 (688-1370) | 820 (569-1139) | 991 (696-1386) | <0.001 |
| Platelets (x109/L) | 206000 (160000-267250) | 172000 (138000-213000) | 209000 (162000-270000) | <0.001 |
| Total bilirrubina (mg/dL) | 0.4 (0.3-0.6) | 0.5 (0.3-0.7) | 0.4 (0.3-0.6) | 0.008 |
| Creatinine (mg/dL) | 0.9 (0.7-1.1) | 1.1 (0.8-1.5) | 0.9 (0.7-1.1) | <0.001 |
| Ferritin (ng/mL) | 868 (443.1-1685.6) | 1047.9 (492.7-1716.7) | 860.7 (432.3-1681.0) | 0.219 |
| Lactate (mmol/L) | 1.4 (1.1-1.9) | 1.5 (1.2-2.1) | 1.4 (1.1-1.9) | 0.001 |
| CRP (mg/L) | 86.0 (46.0-146.1) | 112.0 (64.8-184.1) | 84.0 (44.4-142.0) | <0.001 |
| AST (U/L) | 44.0 (30.0-66.0) | 52.5 (36.0-78.1) | 43.0 (30.0-65.0) | <0.001 |
| ALT (U/L) | 38.0 (24.0-65.0) | 37.0 (24.0-60.0) | 38.0 (24.0-65.1) | 0.444 |
| Urea (mg/dL) | 36.0 (27.0-50.0) | 46.0 (32.0-73.1) | 35.3 (26.0-49.0) | <0.001 |
| Arterial pH | 7.4 (7.4-7.5) | 7.4 (7.4-7.5) | 7.4 (7.4-7.5) | <0.001 |
| Arterial pCO2 | 35.0 (31.5-38.8) | 34.0 (30.6-37.6) | 35.0 (31.6-38.9) | <0.001 |
| Arterial pO2 | 71.0 (61.0-88.0) | 68.0 (57.6-81.8) | 71.6 (61.1-88.6) | <0.001 |
| Bicarbonate (mEq/L) | 23.5 (21.4-25.5) | 22.3 (20.0-24.3) | 23.6 (21.6-25.6) | <0.001 |

^1^Values in numbers (percentage) or medians (interquartile range). ^2^Wilcoxon rank sum test; Fisher's exact test. ALT: alanine aminotransferase; AST: aspartate aminotransferase; CRP: C-reactive protein; KRT: kidney replacement therapy.
