# Supplementary material for: Temporal validation of the MMCD score to predict kidney replacement therapy and in-hospital mortality in COVID-19 patients
Source: BMC Nephrol. 2023 Oct 4;24:292. doi: 10.1186/s12882-023-03341-9 (PMC10552198; doi:10.1186/s12882-023-03341-9)
Supplement: Supplementary file 3 — Additional file 3: Table S3. Clinical manifestations and laboratory findings of the patients hospitalized with COVID-19, considering death and discharge, 2021/2022. [file 12882_2023_3341_MOESM3_ESM.docx]

**Table S3.** Clinical manifestations and laboratory findings of the patients hospitalized with COVID-19, considering death and discharge, 2021/2022.

| **Variables** | **Overall^1^**  (n=9422) | **Death^1^**  (n=1706) | **Discharge^1^** (n=7715) | **p-value^2^** |
| --- | --- | --- | --- | --- |
| *Symptoms* |  |  |  |  |
| Adynamia | 2027 (21.5%) | 378 (22.2%) | 1649 (21.4%) | 0.474 |
| Ageusia | 826 (8.8%) | 125 (7.3%) | 701 (9.1%) | 0.020 |
| Anosmia | 913 (9.7%) | 142 (8.3%) | 771 (10.0%) | 0.037 |
| Headache | 2051 (21.8%) | 263 (15.4%) | 1788 (23.2%) | <0.001 |
| Rhinorrhea | 1185 (12.6%) | 192 (11.3%) | 993 (12.9%) | 0.070 |
| Diarrhea | 1499 (15.9%) | 252 (14.8%) | 1247 (16.2%) | 0.165 |
| Dyspnea | 6228 (66.1%) | 1196 (70.1%) | 5032 (65.2%) | <0.001 |
| Sore throat | 954 (10.1%) | 154 (9.0%) | 800 (10.4%) | 0.101 |
| Fever | 4850 (51.5%) | 771 (45.2%) | 4079 (52.9%) | <0.001 |
| Hyporexia | 1430 (15.2%) | 258 (15.1%) | 1172 (15.2%) | 0.970 |
| Myalgia | 3081 (32.7%) | 465 (27.3%) | 2616 (33.9%) | <0.001 |
| Náusea / Vomiting | 1299 (13.8%) | 200 (11.7%) | 1099 (14.2%) | 0.006 |
| Dry cough | 6383 (67.8%) | 1076 (63.1%) | 5307 (68.8%) | <0.001 |
| *Admission data* |  |  |  |  |
| Hemoglobin (g/dL) | 13.3 (12.1-14.4) | 13.3 (12.0-14.5) | 13.3 (12.2-14.4) | 0.521 |
| White blood cell count (x109/L) | 7530 (5,600-10080) | 7660.0 (5500-10490) | 7505 (5610-10000) | 0.184 |
| Neutrophilis (x109/L) | 5690 (4003-8057) | 6080 (4173-8516) | 5627 (3970-7943) | <0.001 |
| Lymphocytes (x109/L) | 978 (688-370) | 804 (561-153) | 1006 (710-1400) | <0.001 |
| Platelets (x109/L) | 206000 (160000-267500) | 179000 (142000-225000) | 212000 (164000-273000) | <0.001 |
| Total bilirrubina (mg/dL) | 0.4 (0.3-0.6) | 0.5 (0.3-0.7) | 0.4 (0.3-0.6) | <0.001 |
| Creatinine (mg/dL) | 0.9 (0.7-1.1) | 1.0 (0.8-1.4) | 0.9 (0.7-1.1) | <0.001 |
| Ferritin (ng/mL) | 868.0 (443.1-1685.6) | 1208.2 (539.7-2000.0) | 836.5 (427.1-1615.3) | <0.001 |
| Lactate (mmol/L) | 1.4 (1.1-1.9) | 1.6 (1.2-2.2) | 1.4 (1.1-1.9) | <0.001 |
| CRP (mg/L) | 86.0 (46.0-146.1) | 110.8 (63.0-179.0) | 82.4 (43.1-138.8) | <0.001 |
| AST (U/L) | 44.0 (30.0-66.0) | 50.0 (36.0-77.0) | 43.0 (30.0-64.0) | <0.001 |
| ALT (U/L) | 38.0 (24.0-65.0) | 36.0 (23.0-59.0) | 38.0 (24.0-66.4) | 0.009 |
| Urea (mg/dL) | 36.0 (27.0-50.0) | 45.0 (31.0-67.4) | 35.0 (26.0-47.8) | <0.001 |
| Arterial pH | 7.4 (7.4-7.5) | 7.4 (7.4-7.5) | 7.4 (7.4-7.5) | <0.001 |
| Arterial pCO2 | 35.0 (31.5-38.8) | 34.1 (30.7-38.0) | 35.0 (31.7-38.9) | <0.001 |
| Arterial pO2 | 71.0 (61.0-88.0) | 68.0 (57.2-83.8) | 72.0 (61.8-88.8) | <0.001 |
| Bicarbonate (mEq/L) | 23.5 (21.4-25.4) | 22.4 (20.0-24.7) | 23.7 (21.7-25.6) | <0.001 |

^1^Values in numbers (percentage) or medians (interquartile range). ^2^Wilcoxon rank sum test; Fisher's exact test. ALT: alanine aminotransferase; AST: aspartate aminotransferase; CRP: C-reactive protein.
