# Supplementary material for: Temporal validation of the MMCD score to predict kidney replacement therapy and in-hospital mortality in COVID-19 patients
Source: BMC Nephrol. 2023 Oct 4;24:292. doi: 10.1186/s12882-023-03341-9 (PMC10552198; doi:10.1186/s12882-023-03341-9)
Supplement: Supplementary file 4 — Additional file 4: Table S4: Features' importance and contribution to the final model. [file 12882_2023_3341_MOESM4_ESM.docx]

**Table S4:** Features' importance and contribution to the final model.

| **All inpatients^1^** | **Patients in intensive care^2^** | **All hospitalized patients, considering MV anytime during hospital stay^3^** |
| --- | --- | --- |
| Platelets (0.22) | Creatinine (0.24) | MV (0.24) |
| Urea (0.22) | Urea (0.21) | Urea (0.17) |
| Creatinine (0.22) | Platelets (0.2) | Creatinine (0.16) |
| Age (0.18) | Age (0.18) | Platelets (0.16) |
| Sex (0.03) | Sex (0.03) | Age (0.14) |
| MV (0.03) | Modified frailty index (0.03) | Sex (0.02) |
| Hypertension (0.02) | Hypertension (0.02) | Hypertension (0.02) |
| Diabetes mellitus (0.02) | Diabetes mellitus (0.02) | Diabetes mellitus (0.02) |
| Modified frailty index (0.02) | SVNI (0.02) | Modified frailty index (0.02) |
| SVNI (0.02) | Amines (0.02) | SVNI (0.02) |
| Chronic kidney disease (0.01) | Chronic kidney disease (0.01) | Chronic kidney disease (0.01) |
| Glasgow (0.01) | Glasgow (0.01) | Glasgow (0.01) |
| Amines (0.01) | MV (0.01) | Amines (0.01) |

^1^Patient data on hospital admission; ^2^Patient data on admission to the ICU; ^3^Patient data on hospital admission, replacing MV on admission for MV anytime during hospital stay. MV: mechanical ventilation; SVNI: non-invasive ventilatory support (supplementation of oxygen via a face mask or catheter or high-flow nasal oxygen).
